# Genome-wide identification and expression analysis of the *CLC* gene family in pomegranate (*Punica granatum*) reveals its roles in salt resistance

**DOI:** 10.1186/s12870-020-02771-z

**Published:** 2020-12-11

**Authors:** Cuiyu Liu, Yujie Zhao, Xueqing Zhao, Jianmei Dong, Zhaohe Yuan

**Affiliations:** 1 Co-Innovation Center for Sustainable Forestry in Southern China, Nanjing Forestry University, Nanjing, 210037 China; 2 College of Forestry, Nanjing Forestry University, Nanjing, 210037 China

**Keywords:** CLC gene family, Phylogenetic analysis, Anion content, Expression pattern, NaCl stress

## Abstract

**Backgrounds:**

Pomegranate (*Punica granatum* L.) is an important commercial fruit tree, with moderate tolerance to salinity. The balance of Cl^−^ and other anions in pomegranate tissues are affected by salinity, however, the accumulation patterns of anions are poorly understood. The chloride channel (CLC) gene family is involved in conducting Cl^−^, NO_3_^−^, HCO_3_^−^ and I^−^, but its characteristics have not been reported on pomegranate.

**Results:**

In this study, we identified seven *PgCLC* genes, consisting of four antiporters and three channels, based on the presence of the gating glutamate (E) and the proton glutamate (E). Phylogenetic analysis revealed that seven PgCLCs were divided into two clades, with clade I containing the typical conserved regions GxGIPE (I), GKxGPxxH (II) and PxxGxLF (III), whereas clade II not. Multiple sequence alignment revealed that PgCLC-B had a P [proline, Pro] residue in region I, which was suspected to be a NO_3_^−^/H^+^ exchanger, while PgCLC-C1, PgCLC-C2, PgCLC-D and PgCLC-G contained a S [serine, Ser] residue, with a high affinity to Cl^−^. We determined the content of Cl^−^, NO_3_^−^, H_2_PO_4_^−^, and SO_4_^2−^ in pomegranate tissues after 18 days of salt treatments (0, 100, 200 and 300 mM NaCl). Compared with control, the Cl^−^ content increased sharply in pomegranate tissues. Salinity inhibited the uptake of NO_3_^−^ and SO_4_^2−^, but accelerated H_2_PO_4_^−^ uptake. The results of real-time reverse transcription PCR (qRT-PCR) revealed that *PgCLC* genes had tissue-specific expression patterns. The high expression levels of three antiporters *PgCLC-C1*, *PgCLC-C2* and *PgCLC-D* in leaves might be contributed to sequestrating Cl^−^ into the vacuoles. However, the low expression levels of *PgCLCs* in roots might be associated with the exclusion of Cl^−^ from root cells. Also, the up-regulated *PgCLC-B* in leaves indicated that more NO_3_^−^ was transported into leaves to mitigate the nitrogen deficiency.

**Conclusions:**

Our findings suggested that the *PgCLC* genes played important roles in balancing of Cl^−^ and NO_3_^−^ in pomegranate tissues under salt stress. This study established a theoretical foundation for the further functional characterization of the *CLC* genes in pomegranate.

**Supplementary Information:**

The online version contains supplementary material available at 10.1186/s12870-020-02771-z.

## Background

Pomegranate (*Punica granatum* L.), a salt-tolerant plant, is widely grown in the arid and semiarid regions, where is always suffering the soil salinization [1]. Bhantana et al. [2] reported that pomegranate could be used as a model plant for deciduous fruit trees to study the responses to environmental stresses. In our previous study, we found that the Cl^−^ content was two times more than the Na^+^ content in pomegranate tissues, and uptake of other anions was also affected by various concentration of salinity [3]. Chlorine is an essential micronutrient for plants, predominantly occurring in the form of Cl^−^ [4, 5]. It is mainly involved in plant physiological activities, such as photosynthesis, regulation of stomatal opening and closing, stabilization of the membrane potential, regulation of intracellular pH gradients and electrical excitability [5]. Excess and/or deficiency of Cl^−^ leads to weak plant growth, low yield and poor quality [6, 7]. In a salinized environment, mostly caused by high NaCl, the foliar salt damage of some plants was mainly caused by Na^+^ [8], while that of other plants, such as tobacco (*Nicotiana tabacum*) [7], grape (*Vitis vinifera*) [9], citrus (*Citrus aurantium*) [10] and soybean (*Glycine max*) [11, 12] was mainly caused by Cl^−^. Previous researches reported that the accumulation patterns of anions, such as Cl^−^, NO_3_^−^, HCO_3_^−^, and SO_4_^2−^ in plant tissues were associated with the plant salt tolerance [6]. Also, the NO_3_^−^/Cl^−^ even equal to the K^+^/Na^+^, which was confirmed as one of the critical determinants of plant salt resistance [8, 13]. Therefore, the study on the underlining mechanisms between uptake and transport of Cl^−^ and other anions in pomegranate tissues and salinity conditions was contributed to elucidate the pomegranate salt tolerance.

Chlorine channel (CLC) proteins are highly associated with uptake and transport of these anions, like Cl^−^, NO_3_^−^, HCO_3_^−^, I^−^, and Br^−^ [14–17]. The first CLC family gene (*CLC-0*) was identified from the electric organ of marine ray (*Torpedo marmorata*) [18], and since then, some new members have been found in bacteria, yeast, mammals and plants [19]. In land plants, the first CLC gene, *CLC-Nt1*, was cloned in tobacco [20]. Subsequently, numerous *CLC* gene homologues were isolated from *Arabidopsis* [21], rice (*Oryza sativa*) [22], soybean (*Glycine max*) and trifoliate orange (*Poncirus trifoliata*) [23], etc. All of the CLC proteins have a highly conserved voltage-gated chloride channel (Voltage-gate CLC) domain and two CBS (cystathionine beta synthase) domains of putative regulatory function [14]. Also, the CLC gene family members contain three highly conserved regions related to anion selectivity: GxGIPE (I), GKxGPxxH (II) and PxxGxLF (III) [24]. If the x residue in the conserved region (I) is P [proline, Pro], NO_3_^−^ is preferentially transported, whereas if it is substituted by S [serine, Ser], Cl^−^ is preferentially transported [25]. The first x residue in conserved region II and the next fourth residue of the conserved region III can both be E (Glu) residue, which are signatures for CLC antiporters [26, 27]. However, if any other amino acids are found at these positions, such as in AtCLCe, AtCLCf and AtCLCg, these proteins may exert CLC channels activity [27]. Therefore, CLC proteins may act as Cl^−^ channels or as Cl^−^/H^+^-exchangers (antiporters) [19]. The Cl^−^ channels mediate passive transport by dissipating pre-existing electrochemical gradients, while the antiporters mediate active transport by coupling with energy consumption to move the substrate against an electrochemical gradient [27]. In higher plants, CLC proteins play vital roles in the control of electrical excitability, turgor maintenance, stomatal movement, ion homeostasis, as well as in responses to biotic and/or abiotic stress [28–30].

In *Arabidopsis*, there are seven reported CLC genes: *AtCLCa* ~ *AtCLCg*, which play different roles in diverse cell organelles [28, 31]. Barbierbrygoo et al. [32] and Marmagne et al. [33] suggested that AtCLCa ~ AtCLCd and AtCLCg were clustered into a distinct branch, belonging to eukaryotic CLCs, while AtCLCe and AtCLCf are closely related to prokaryotic CLC channels. *AtCLCa* codes for an NO_3_^−^/H^+^ exchangers localized in the vacuolar membrane, which is critically involved in this nitrate accumulation in the vacuole [21]. *AtCLCb*, coding for a vacuolar antiporter, shares 80% identity with *AtCLCa*, is highly expressed in young roots, hypocotyl and cotyledons [34]. *AtCLCc* is essential for the detoxification of cytosol by sequestrating Cl^−^ into the vacuoles under salt stress, and it is strongly expressed in guard cells, pollen and roots [28]. AtCLCd and AtCLCf, both localized in Golgi membranes, may play a role in the acidification of the *trans*-Golgi vesicles network [31, 33], while AtCLCe is targeted to the thylakoid membranes in chloroplasts [33]. *AtCLCg*, the closest homolog to *AtCLCc* (62% identity), plays a physiological role in the Cl^−^ homeostasis during NaCl stress [35]. In other plants, many *CLC* genes are involved in anions transport and in the response to salt stress. For instance, the expression level of *OsCLC-1* is upregulated in rice under NaCl stress [22]; *PtrCLC* genes are profoundly induced in orange by salt stress [23]; *GmCLC1* has been found to enhanced salt tolerance in transgenic *Arabidopsis* seedlings by reducing the Cl^−^ accumulation in shoots [36]; and *GsCLC-c2* over-expression contributes to Cl^−^ and NO_3_^−^ homeostasis, and therefore confers the salt tolerance on wild soybean [37].

However, the characteristics of the *CLC* genes have not been reported on pomegranate. Therefore, this study made a comprehensive, genome-wide inventory of the CLC gene family in pomegranate. In order to reveal the accumulation patterns of Cl^−^ and other anions in pomegranate tissues and the roles of *PgCLCs* in uptake and transport of these anions, we also determined the anions contents and the expression levels of *PgCLCs* in pomegranate tissues under different NaCl concentration, which would comprehensively illuminate the accumulation patterns of anions under NaCl stress, and provide a reference for the further study on functions of the *CLC* gene.

## Results

### Identification of the *CLC* genes in pomegranate

A HMM profile was used to identify the putative *CLC* genes in pomegranate genome. All seven putative *CLC* genes contained a highly conserved Volgate_CLC domain and two CBS domains, and they were named *PgCLC-B* to *PgCLC-G* according to the homologous *AtCLCs* (Table 1). The analysis of protein sequences showed that the PgCLCs contained 698 ~ 797 amino acids and had molecular weights of 75.7 ~ 87.9 kDa. The predicted isoelectric points (pI) of all the PgCLC proteins ranged from 5.86 to 8.44. The grand average of the hydrophobicity (GRAVY) values were all positive values, indicating that the PgCLCs were hydrophobic proteins. There were a number of transmembrane helices (TMHs) in the PgCLCs, ranging from 9 to 11, which were associated with the ion transport.

**Table 1:** Characteristics of the *CLC* genes in pomegranate

Gene ID	Name	Length	Mw (kDa)	pI	GRAVY	Orthologs	TMHs
*CDL15_Pgr005627*	*PgCLC-B*	797	87.9	6.49	0.259	*AtCLC-B*	9
*CDL15_Pgr027626*	*PgCLC-C1*	698	75.7	7.53	0.364	*AtCLC-C*	10
*CDL15_Pgr013895*	*PgCLC-C2*	717	78.1	5.92	0.325	*AtCLC-C*	10
*CDL15_Pgr008552*	*PgCLC-D*	788	86.9	8.57	0.175	*AtCLC-D*	11
*CDL15_Pgr019810*	*PgCLC-E*	764	81.3	5.86	0.188	*AtCLC-E*	10
*CDL15_Pgr012201*	*PgCLC-F*	765	81.7	6.54	0.035	*AtCLC-F*	11
*CDL15_Pgr015371*	*PgCLC-G*	709	77.3	8.44	0.468	*AtCLC-G*	10

Note: molecular weight (Mw), isoelectric points (pI), grand average of the hydrophobicity (GRAVY), transmembrane helices (TMHs)

### Phylogenetic analysis of the CLC gene family in pomegranate

**Fig. 1:**
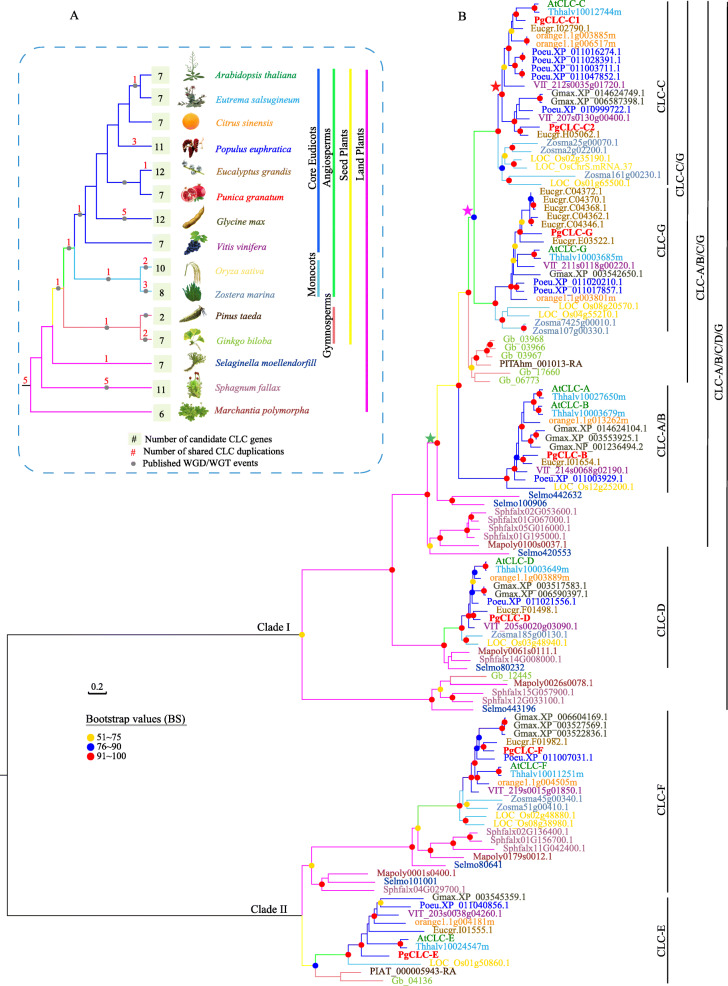
Phylogenetic analysis of the CLC gene family in land plants. **a** Species tree with different branch colors showing distinct species. **b** Phylogenetic tree of the CLC gene family in land plants presented in various branch colors, as in Fig. **a**. Node support (pots) was quantified by aLRT statistics with the SH-like procedure. Colored stars are gene duplication events

To elucidate the evolutionary traits of the CLC gene family in land plants, we investigated 15 interesting species that had available reference genome sequences. Our results showed two obvious clades of the CLC gene tree, clade I was the major group bearing a moderate support (BS = 61%, Fig. S1) and clade II contained two subgroups (Fig. 1). PgCLC-E and PgCLC-F were belonged to clade II and other PgCLCs were belonged to clade I. The divergence of clades I and II might have occurred before the origin of land plants due to each clade consisting of taxa from embrophytes (Fig. 1). Phylogenetic analyses indicated multiple rounds of ancient gene expansion (Fig. 1). The diversity of gene copy number from different lineages (Fig. 1a). The gene tree-species tree reconcilably identified a gene duplication (the red star in Fig. 1b) with a strongly supported (BS = 100, Fig.S1) topology of (core eudicots, core eudicots), which was contributed to the duplication between PgCLC-C1 and PgCLC-C2. A gene duplication (the purple star in Fig. 1b) resulting in a topology of ((core eudicots, monocots), (core eudicots, monocots)) was identified as one duplicate shared by angiosperms, which was associated with the duplication between PgCLC-C and PgCLC-G. Our phylogenetic analyses also found gene expansion in seed plants, with a gene birth from an ancient gene duplication (the green star in Fig. 1b) and a subsequent gene death. The tree topology [(angiosperms, gymnosperms) angiosperms] of the CLC-A/B/C/G genes (Fig. 1) exhibited a gene loss event in gymnosperms. There were two members from *Arabidopsis and Eutrema* in the CLC-A/B subfamily, while only one member PgCLC-B from pomegranate.

Here, our phylogenetic results showed that seven putative *PgCLC* genes originated before the divergence of land plants and were retained after experiencing six times of duplications, including at least one ancient core eudicots-specific duplication (PgCLC-C1 and PgCLC-C2) and one angiosperm-specific expansion (PgCLC-C1/C2 and PgCLC-G) (Fig. 1, Fig. S1).

### Conserved motifs and residues of the CLC gene family

**Fig. 2:**
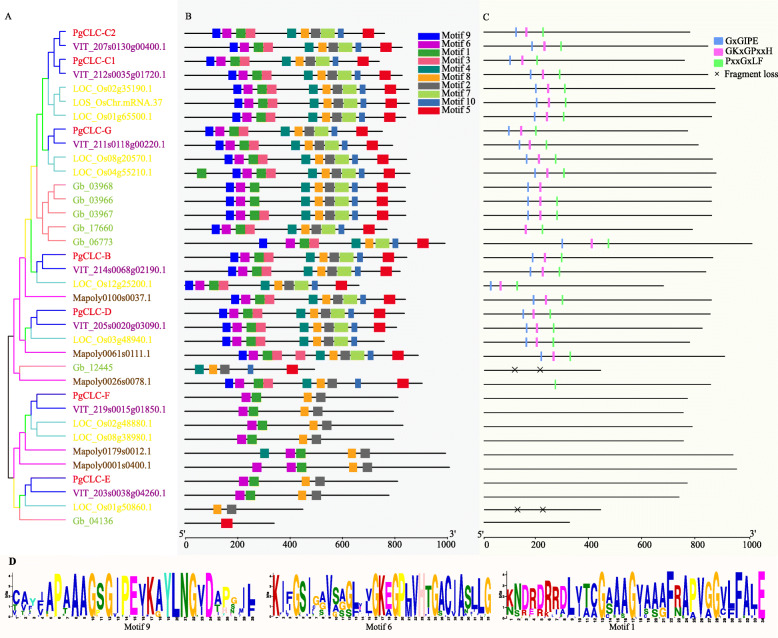
Phylogenetic tree of five species: *Punica granatum*, *Vitis vinifera*, *Oryza sativa*, *Ginkgo biloba* and *Marchantia polymorpha* **a**, motif distribution of CLC proteins **b**, three conserved regions in CLCs **c** and three typical motif logos **d**. Three colored regions are present with their counterpart light-colored motifs, respectively

To further investigate the structural diversity of all CLCs in land plants, the conserved motifs and regions were analyzed. Here, a total of ten motifs were selected, referring as motif 1–10, and five representative species of each taxa were shown (Fig. 2b, Fig. S1B). Different motif patterns were clearly observed in the two clades, as mentioned above (Fig. 1b). For clade I, most of the CLCs possessed ten motifs (Fig. 2b, c; Fig. S2). For clade II, most of the CLC-E and CLC-F proteins possessed four motifs: 6, 1, 8 and 2, which were shared by all of the CLCs of clade I. Three conserved regions GxGIPE (I), GKxGPxxH (II) and PxxGxLF (III) were included in motif 9, motif 6 and motif 1, respectively (Fig. 2b, c and d). Three highly conserved regions of the CLC gene family were shared by members of clade I, whereas they were not shared by members of clade II (Fig. 2b, c; Fig. S2).

**Fig. 3:**
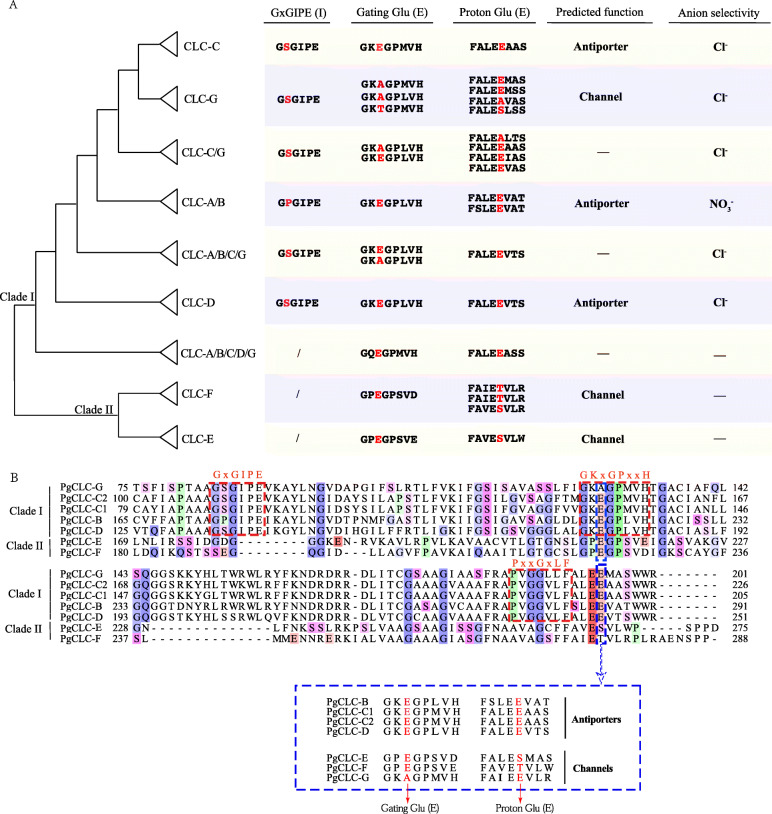
Conserved residues distribution in the CLC gene family. **a** CLC gene tree with collapsed branches, and the conserved residues of each subfamily are listed on the right. Distinct residues are highlighted in red. **b** Partial sequence alignment of the seven CLC proteins in pomegranate. The conserved regions: GxGIPE (I), GKxGPxxH (II) and PxxGxLF (III) are circled in red color. Conserved E (Glu) residues are circled in blue color. The presence of the conserved gating glutamate (E) and/or proton glutamate (E) residues is a signature for distinguishing the CLC antiporters and CLC channels, respectively

Additionally, to meticulously analyze the conserved regions of CLC proteins, multiple sequence alignment was performed. Members of the CLC-A/B subfamily had a P [proline, Pro] residue in the conserved region GxGIPE (I), while other proteins of the CLC-C, CLC-G and CLC-D subfamilies in clade I had a S [serine, Ser] residue in the conserved region I (Fig. 3a). These critical residues were recognized to have a close relation with anion selectivity. The P [proline, Pro] preferentially transported NO_3_^−^, whereas the S [serine, Ser] preferentially transported Cl^−^ (Fig. 3a). Thus, PgCLC-B was likely a NO_3_^−^/H^+^ exchanger that mainly transported NO_3_^−^, while PgCLC-C, PgCLC-D and PgCLC-G might preferentially transported Cl^−^. The presence of the conserved gating glutamate (E) in conserved region (II) and the proton glutamate (E) residues in the next fourth residue of the conserved region (III) were signatures for CLC antiporters. Otherwise, the conserved gating glutamate (E) of the CLC-G subfamily and the proton glutamate (E) residue of the CLC-E and CLC-F subfamilies were substituted by other amino acids (Fig. 3a), which suggested that the members of these three subfamilies might be CLC channels. Based on these results, we assumed that four PgCLC proteins (PgCLC-B, PgCLC-C1, PgCLC-C2 and PgCLC-D) were CLC antiporters, while the other three PgCLCs (PgCLC-E, PgCLC-F and PgCLC-G) were likely CLC channels (Fig. 3a, b).

### Growth characteristics and anion contents in pomegranate tissues

With the increasing concentration of salinity, dry weights of roots and stems showed no significant changes among each treatment (Table S4, *p <* 0.05). While leaf dry weight and total dry weight first increased and then decreased, reaching a peak at 100 mM salinity level.

**Fig. 4:**
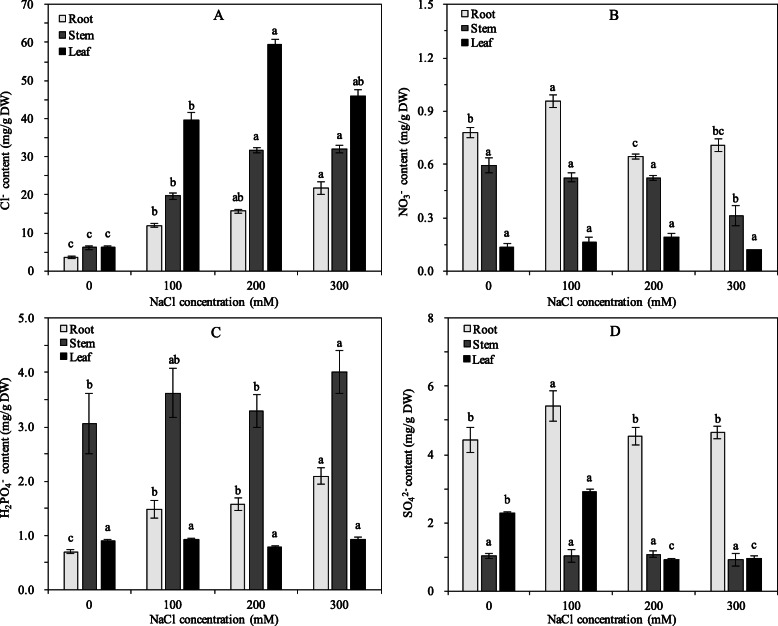
The concentrations of Cl^−^ (**a**), NO_3_^−^ (**b**), H_2_PO_4_^−^ (**c**), and SO_4_^2−^ (**d**) in pomegranate tissues under NaCl stress. The values are the means ± SE of three replicates. Bars with different letters within each panel are significant differences at *p* < 0.05 among different concentrations of salinity according to the Turkey’s test

As shown in Fig. 4a, the contents of Cl^−^ in pomegranate roots, stems and leaves significantly increased with the increasing concentration of NaCl (*p <* 0.05). Under 300 mM NaCl stress, the levels of Cl^−^ in roots, stems and leaves increased 6.19, 5.29 and 7.42 times, compared with control, respectively. The contents of Cl^−^ in plant tissues was ranked as leaf > stem > root. Compared to control, the NO_3_^−^ contents in roots first increased and then decreased, with the highest value at 100 mM salinity. However, the NO_3_^−^ contents in stems and leaves had no obvious changes, except NO_3_^−^ content in stem at 300 mM salinity (*p <* 0.05). The NO_3_^−^ contents in plant tissues was ranked as root > stem > leaf (Fig. 4b). By contrast, the H_2_PO_4_^−^ contents in roots increased along with the increasing salinity, while no significant changes were observed in most leaf and stem samples (*p <* 0.05). Moreover, we found that H_2_PO_4_^−^ was mainly accumulated in stems (Fig. 4c). For the SO_4_^2−^ contents, trends of first increasing and then decreasing in pomegranate roots and leaves were observed with peaks at 100 mM salinity. As Fig. 4d shown, SO_4_^2−^ mainly accumulated in roots, and the content of SO_4_^2−^ in leaves fell sharply under higher salinity (> 200 mM NaCl).

### Expression patterns of the *PgCLC* genes under NaCl stress

**Fig. 5:**
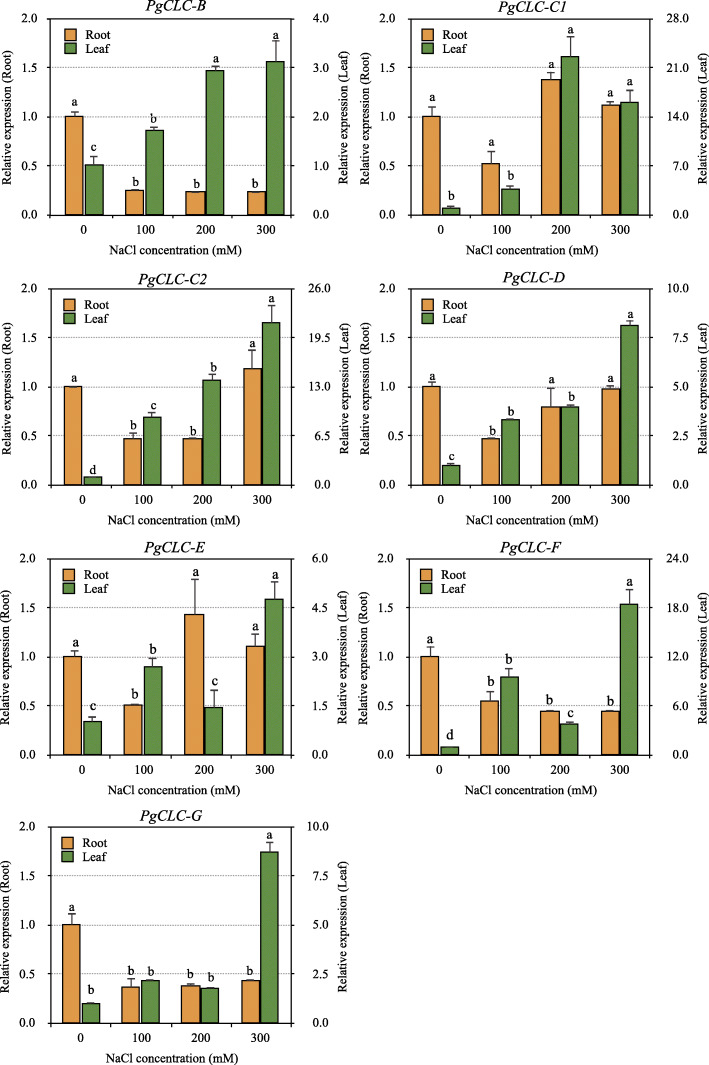
qRT-PCR analysis of the *CLC* genes in pomegranate roots and leaves after 18 days of NaCl stress, the expressional levels are calculated by the 2^−ΔΔCT^ method. Bars with different letters within each panel are significant differences at *p* < 0.05 among different concentrations of salinity according to the Turkey’s test

To further investigate the expression patterns of the *PgCLC* genes, we performed the qRT-PCR analysis in pomegranate roots and leaves. The results showed that all the *PgCLC* genes had tissue-specific expression patterns, with high expression levels in leaves and low expression levels in roots (Fig. 5). Notably, when plants were subjected to salinity, the expression levels of all the tested *PgCLCs* were up-regulated in pomegranate leaves, but were down-regulated or not obviously changed in roots (*p* < 0.01). For instance, the relative expression levels of *PgCLC-B*, *PgCLC-C1*, *PgCLC-C1* and *PgCLC-D* in leaves increased with the increasing salinity; meanwhile, those of *PgCLC-E*, *PgCLC-F* and *PgCLC-G* in leaves significantly increased at high salinity (200 mM). Also, the expression levels of *PgCLC-B*, *PgCLC-F* and *PgCLC-G* in roots decreased and those of *PgCLC-C1*, *PgCLC-C2*, *PgCLC-D* and *PgCLC-E* in roots first decreased at 100 mM salinity level and then recovered slightly at 200 mM and/or 300 mM salinity levels (Fig. 5). Under 300 mM NaCl stress, the expression levels of *PgCLC-C1*, *PgCLC-C2* and *PgCLC-F* in leaves increased by more than 16-fold relative to those of controls.

### Correlation between the anion contents and expression levels of the *PgCLC* genes

**Fig. 6:**
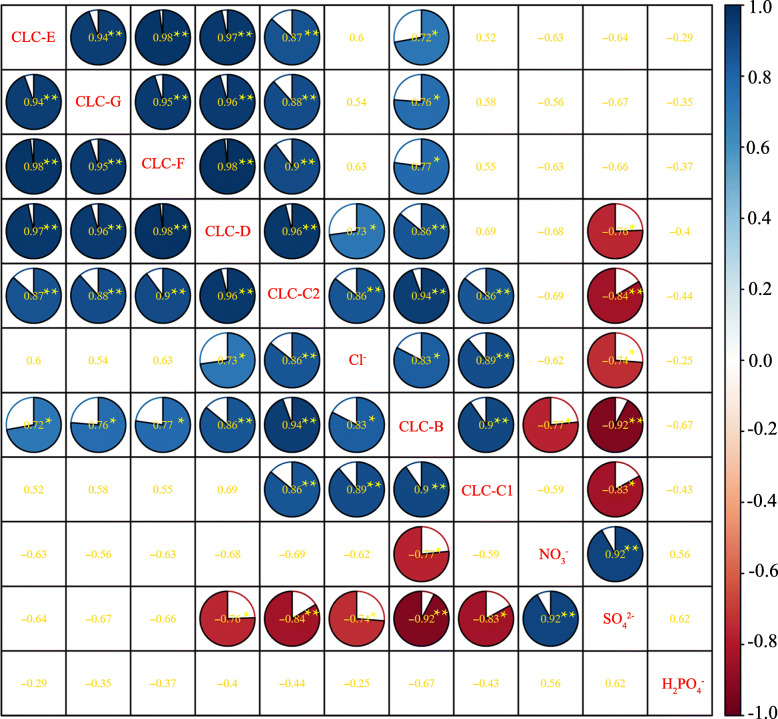
Correlations between the expression levels of seven *PgCLCs* and the anion contents of pomegranate roots and leaves. The blue pie indicates a positive correlation, and the red pie indicates a negative correlation. The darker the color, the more significant the correlation. The gold numbers are correlation coefficients. ** is highly significant at *p* < 0.01 and * is significant at *p* < 0.05

Correlation analysis showed that the *PgCLC* genes were positively correlated with each other (Fig. 6, *p* < 0.05). The Cl^−^ contents had significantly positive correlations with *PgCLC-B*, *PgCLC-C1*, *PgCLC-C2* and *PgCLC-D*, while the SO_4_^2−^ content had significantly negative correlations with these genes. Meanwhile, the contents of Cl^−^ and SO_4_^2−^ were negatively correlated with each other (*p* < 0.05). A significantly negative correlation between the NO_3_^−^ content and the expression level of *PgCLC-B*, and a significantly positive correlation between the SO_4_^2−^ were found. There was no significant relationship between the H_2_PO_4_^−^ content and the other indexes (Fig. 6). These findings suggested that accumulation of Cl^−^, SO_4_^2−^ and NO_3_^−^ in pomegranate tissues was associated with the expression levels of the *PgCLC* genes under salt stress.

## Discussion

### Identification of the CLC gene family in pomegranate

The CLC gene family is an evolutionarily well-conserved family, which has been found in prokaryotes and eukaryotes [14, 19]. CLC channels form two-pore homodimers with two monomers, and each monomer has its own pore through which Cl^−^ and other anions (HCO_3_^−^, I^−^, and NO_3_^−^) may be conducted [14, 38]. CLC transporters and channels have regulatory functions when ATP, ADP, AMP, or adenosine are bound at the CBS domains [38]. In pomegranate, each CLCs contains one voltage-gated CLC domain near the N-terminus and two CBS domains at the C-terminus. The specific effect implies that individual CLC transporters and channels are sensitive to the cell’s metabolic state [14, 27].

### Phylogenic relationships of the CLC gene family

Numerous early whole-genome duplication (WGD) events in plants, including the gamma event shared by core-eudicots [39], the WGD event shared by angiosperms [40–42], and the seed-plant WGD event [40, 41], contribute to gene duplications. The diversity of gene copy numbers from different lineages (Fig. 1a) might be related to the rounds of WGD events shared with the taxon [43]. Based on the phylogenic analysis of the CLC gene family in 15 land plants, seven PgCLCs were divided into two clades, with clade I belonging to a eukaryotic branch and clade II belonging to a bacterial branch [32, 33]. The divergence of clade I and clade II might have occurred before the origin of land plants due to each clade consisting of taxa from embrophytes (Fig. 1). The CLCs topology was consistent with that of *Arabidopsis* [32, 33], tobacco [44], tea (*Camellia sinensis*) [24] and trifoliate orange [23]. Phylogenetic analysis also indicated multiple rounds of ancient gene expansion (Fig. 1). For example, the gene duplication between PgCLC-C1 and PgCLC-C2 (the red star in Fig. 1b) was supported by the duplication burst shared by core eudicots [45]. The gene duplication between the CLC-C and CLC-G subfamilies was due to one duplicate shared with angiosperms (the purple star in Fig. 1b) [45]. In the CLC-A/B subfamily, only one member PgCLC-B was identified in pomegranate. While there were two members from *Arabidopsis and Eutrema* due to a specific gene duplication shared by plants of Brassicaceae [46]. Our phylogenetic analyses also found a gene expansion in seed plants, with a gene birth from an ancient gene duplication (the green star in Fig. 1b) and a subsequent gene death. The CLC-A/B/C/G subfamily (Fig. 1) exhibited a gene loss event in gymnosperms after experiencing the seed-plant WGD event [40, 41] despite the fact that the absence of the gene might have resulted from the putative incompleteness of the genome assembly and annotation. Recent phylogenetic studies have also found land plant-scale gene birth and expansion, such as in the CYP75 gene family [43] and GH28 gene family [40].

Additionally, the distributions of conserved motifs and conserved regions were consistent with the phylogenetic relationships (Fig. 2). Three highly conserved regions of the CLC gene family, GxGIPE (I), GKxGPxxH (II) and PxxGxLF (III) were shared by the members of clade I, whereas they were not shared by the members of clade II. This finding indicated that the divergence of clades I and clade II might be due to the presence of these conserved regions (Fig. 2c). Our study also found that the x residue in the conserved region (I) of the CLC-A/B subfamily was P [proline, Pro] (Fig. 3; Fig. S2), which preferentially transports NO_3_^−^ [25], while that of the CLC-C, CLC-D and CLC-G subfamily was S [serine, Ser] (Fig. 3; Fig. S2), which preferentially transports Cl^−^ [25]. Thus, PgCLC-B was likely a NO_3_^−^/H^+^ exchanger that mainly transported NO_3_^−^ rather than Cl^−^ [21, 34], while PgCLC-C, PgCLC-D and PgCLC-G might have high affinity for Cl^−^ [21, 34]. A presence of the gating glutamate (E) residue and proton glutamate (E) residue was signature for CLC antiporters [26, 27]. However, if one or both of the Glu (E) residues were substituted by any other amino acids in the conserved region, the CLC proteins might exhibit CLC channels activity [27]. Therefore, we assumed that PgCLC-B, PgCLC-C1, PgCLC-C2 and PgCLC-D were CLC antiporters and PgCLC-E, PgCLC-F and PgCLC-G were CLC channels. Our results were in line with the findings in *Arabidopsis* [26, 27].

### *PgCLCs* played roles in response to NaCl stress

As an essential micronutrient for plants, Cl^−^ is beneficial for plants at low concentrations in media [4, 5]. However, high salinity (mainly NaCl) may cause a perturbation of Na^+^ and Cl^−^ at both the cellular and whole plant levels, which affects the uptake and transport of other mineral ions, such as K^+^, Ca^2+^, Mg^2+^, H_2_PO_4_^−^, NO_3_^−^ and SO_4_^2−^ [47, 48]. In this study, we focused on the anion accumulation in pomegranate tissues. CLC proteins are expressed on the cell membrane and conduct Cl^−^ or other anions, such as HCO_3_^−^, I^−^, and NO_3_^−^ [14–17]. Compared with the controls, the expression levels of *PgCLCs* were up-regulated in leaves and down-regulated or not significantly changed in roots (*p* < 0.01). The tissue-specific expression of seven *PgCLCs* indicated different mechanisms of transporting anions in pomegranate roots and leaves. Our study found that the Cl^−^ contents in pomegranate tissues sharply increased with an order of leaf > stem > root (Fig. 4a), indicating a relatively strong ability for pomegranate to transport and accumulate toxic ions in the acrial parts [49]. In leaves, the high expression levels of *PgCLCs* suggested the inclusion of Cl^−^ into leaf cells or organelles. Individually, the expression levels of three antiporters *PgCLC-C1*, *PgCLC-C2* and *PgCLC-D* were significantly positive with the Cl^−^ content, which might be contributed to the sequestration of Cl^−^ into the leaf vacuoles [28, 50]. However, the low expression levels of *PgCLCs* in roots suggested the exclusion of Cl^−^ from root cells. The recovery of *PgCLC-C1*, *PgCLC-C2* and *PgCLC-D* in roots were contributed to the sequestration of Cl^−^ into the root vacuoles at high salinity levels [28, 50]. Therefore, *PgCLCs* were supposed to alleviate the deleterious effects of Cl^−^ via excluding the Cl^−^ from root cells and sequestrating Cl^−^ into the leaf vacuoles [28, 50]. Similarly, some halophytes prefer to transport and accumulate detrimental ions in the acrial parts under salt stress [51, 52]. On the other hand, under moderate salinity (≤ 200 mM NaCl), the low expression levels of three Cl^−^ channels *PgCLC-E*, *PgCLC-F* and *PgCLC-G* in leaves (Fig. 5), suggested the capacity for pomegranate to inhibit the Cl^−^ influx into cells or organelles [3, 33].

Also, the NO_3_^−^ contents of pomegranate roots first increased and then decreased, and that of leaves not changed under salt stress (Fig. 4b, d). The increase of the Cl^−^ content was concomitant with the decrease of the NO_3_^−^ content in pomegranate tissues, which could be due to the antagonism between Cl^−^ and NO_3_^−^ [53]. The expression level of *PgCLC-B* (a NO_3_^−^/H^+^ exchanger) [21, 34], was significantly positive correlated with the Cl^−^ content, and significantly negative correlated with the NO_3_^−^ content (*p* < 0.05). These results suggested that the decreased uptake of NO_3_^−^ in roots might be due to the inhibition of *PgCLC-B* activity under salt stress [21, 34]. The inhibition of nitrogen uptake was also associated with nitrate transporter (NRTs) [54, 55]. Meanwhile, the increased expression level of *PgCLC-B* in leaves indicated an acceleration of transporting NO_3_^−^ into leaves to mitigate the nitrogen deficiency [34]. Teakle et al. [6] reported that the increased concentration of NO_3_^−^ in media reduced the Cl^−^ content in leaves and then mitigated the foliar salt damage, the NO_3_^−^/Cl^−^ was contributed to the plant salt resistance [8, 13]. In pomegranate, it was observed that a low ratio of NO_3_^−^/Cl^−^ might cause a reduction in growth [56] (data not showed).

In a word, these findings suggested that the *PgCLC* genes played important roles in uptake and transport of Cl^−^ and NO_3_^−^ in pomegranate tissues under salt stress [15–17, 28]. While the accumulation pattern of SO_4_^2−^ was associated with the other genes, such as sulfate transporters [57]. Wei et al. [23] found that *PtrCLC* genes were dramatically induced in response to NaCl stress, and *PtrCLC6* showed a leaf-specific expression pattern in trifoliate orange. Zhang et al. [44] observed that all of the expressed *NtCLC* genes had a low expression level in tobacco roots under salt stress. Our findings are consistent with these reports. In addition, the functional characterization of each *PgCLC* genes need to further study.

## Conclusions

In this study, we identified and characterized seven *CLC* genes in pomegranate. Phylogenetic analysis indicated that the PgCLCs were divided into two distinct clades, with a similar distribution of conserved motifs and regions in the members of each clade. In pomegranate, the *PgCLC* genes displayed a tissue-specific expression pattern, with the high expression levels in leaves and the low expression levels in roots under salt stress. *PgCLCs* were supposed to play important roles in balancing of Cl^−^ and NO_3_^−^ in pomegranate tissues under salt stress. Our study provides the basis for the further functional characterization of the *PgCLC* genes.

## Methods

### Identification of the CLC gene family in pomegranate

A Hidden Markov Model (HMM) profile of the voltage-gated chloride channel (Voltage-gate CLC) domain (Accession no. PF00654) was employed to identify the putative CLC proteins from genome sequences using the software HMMER v3.1b1 [58] accorrding to the methods of Zhang et al. [43] with a cut-off E-value of ≤1e^− 10^. To construct a representative phylogeny across land plants, ten angiosperms (eight core eudicots and two monocots), two gymnosperms and three bryophytes were selected, including *Arabidopsis thaliana*, *Citrus sinensis*, *Eucalyptus grandis*, *Eutrema salsugineum*, *Glycine max*, *Populus euphratica*, *Punica granatum*, and *Vitis vinifera* as the core eudicots; *Oryza sativa* and *Zostera marina* as monocots; *Ginkgo biloba* and *Pinus taeda* as gymnosperms; and *Marchantia polymorpha*, *Selaginella moellendorffii* and *Sphagnum fallax* as bryophytes. Seven CLC proteins from *Arabidopsis thaliana* were obtained from the Arabidopsis Information Resource (TAIR) (http://www.arabidopsis.org/). The genome sequences of 14 other species were downloaded from URLs (Table S1). Firstly, all the putative CLC proteins were identified from the genomes of the 14 species. Subsequently, the CLC candidates were manually curated, and the nonredundant CLC proteins were further analyzed using the NCBI Conserved Domain Database (CDD, http://www.ncbi.nlm.nih.gov/cdd/) and SMART programs (http://smart.embl-heidelberg.de/) to confirm the presence of the Voltage-gate CLC domain. The theoretical isoelectric point (pI), molecular weight (Mw) and grand average of hydrophobicity (GRAVY) of seven PgCLC proteins were predicted using the Prot-Param tool (http://web.expasy.org/protparam/). The number of transmembrane helices (TMHs) was predicted using TMHMM Server v. 2.0 (http://www.cbs.dtu.dk/services/TMHMM/) and TMpred (https://embnet.vital-it.ch/software/TMPRED_form.html).

### Phylogenetic analysis of CLC gene family

To estimate the origin and divergence of CLC genes, an maximum likelihood (ML) tree of these genes was reconstructed using iQ-TREE and used to map on a species tree of land-plants, which is a part of the tree of life as inferred in the OneKP project [40], by using the methods in Zhang et al [59]. All of the puative CLC proteins were aligned using MUSCLE v3.8.31 [60] with the ‘auto’ setting. To improve the valid phylogeny signals, the low-quality alignment regions and incorrect sequences with apparent splice variants were removed [61]. Finally, a total of 113 putative CLC candidates were retained, including seven PgCLCs (Table S2). The conserved blocks were retained by Gblocks v0.91b [62], and then, phylogenetic analysis was preformed using iQ-TREE v2 [63] with the LG + R6 model, 1000 bootstraps, and the Shimodaira-Hasegawa-like aLRT (SH-aLRT) test. Putative functional homologs were identified from a gene clade that contained the query gene from *Arabidopsis* and was likely derived from an ancestral gene from land plants [59].

### Conserved motifs and residues prediction of CLC proteins

The conserved motifs and regions of all CLC proteins were predicted by the MEME tool (http://meme-suite.org/tools/meme). The maximum number of motifs was set to 10, and the optimum motif width was ≥ 6 and ≤ 50. Three conserved regions (GxGIPE (I), GKxGPxxH (II) and PxxGxLF (III)) of the CLC gene family were searched by the MAST tool (http://meme-suite.org/tools/mast) with a sequence threshold ≤30 and an E-value ≤1e^− 10^ for motifs. Multiple sequence alignment of CLCs was performed by Clustal X v2.0 [64] and visualized by Jalview v1.0 [65].

### Plant materials and growth conditions

Pomegranate cv. ‘Taishanhong’ cuttings (one-year-old, collected from Pomegranate repository of Nanjing Forestry University. China) were planted in a phytotron for six months, with a 28/22 °C day/night temperature, 60% humidity and 14 h light/10 h dark photoperiod. Hoagland’s nutrient solution [66] was supplied at the beginning of the experiment. A total of 24 pots (one plant per plot) were arranged in a completely randomized 3 blocks, and 8 pots per block, and every 2 pots were designed as a biological replicate. All plants were fertigated with half-strength Hoagland’s solution containing 0 (control), 100, 200, or 300 mM NaCl every six days, respectively. A saucer was placed under the containers to keep the soil moist. According to our previous study, after 18 days of treatments, the salt damage on pomegranate plant were significant [3]. Therefore, we harvested all plants separately to collect roots, stems, and leaves after 18 d.

### Anion content measurement

The dry weights of pomegranate roots, stems and leaves were determined after drying in a heating oven at 75 °C for 48 h. Dry samples were finely milled to pass through a 40-mesh sieve. Then 0.4 g of samples were treated with 50 mL of deionized water for 1 h in an ultrasonic extractor at room temperature, and then the obtained extracts were used to determine the contents of Cl^−^, NO_3_^−^, H_2_PO_4_^−^, and SO_4_^2−^ using an ion chromatography (ICS900 ion chromatographic system; AS4A-SC ion-exchange column, CD M-II electrical conductivity detector, mobile phase: Na_2_CO_3_/NaHCO_3_ = 1.7/1.8 mM; Dionex, Sunnyvale, USA) [67].

### Expression levels of *PgCLCs* by quantitative real-time PCR (qRT-PCR)

Total RNA was extracted from fresh root and leaf samples using the BioTeke plant total RNA extraction kit (BioTeke Corporation, Beijing, China) according to the manufacturer’s instructions. First-strand cDNA was prepared using a reverse transcription kit-PrimeScript™ RT reagent Kit with gDNA Eraser (TaKaRa Bio Tech Co., Ltd., Beijing, China). The primers of seven *PgCLCs* were designed with NCBI primer-BLAST (Table S3). Real-time RT-PCR (qRT-PCR) was performed using a 7500 fast Real-Time PCR system (Applied Biosystems, CA, USA) with three biological and three technical replicates for each cDNA sample, and the results were quantitatively analyzed by the 2^−ΔΔCT^ method [68]. Each reaction was carried out in a final volume of 20 μL, containing 10 μL of TB Green *Premix Ex Taq*, 0.4 μL of ROX Reference Dye II, 0.8 μL of upstream/downstream primers, 1 μL of cDNA template and 7 μL of ddH_2_O. The PCR thermal cycler was set as follows: pre-denaturation at 95 °C for 30 s; 40 cycles of 95 °C for 5 s and 60 °C for 34 s; the dissociation stage was set as follows: 95 °C for 15 s, 60 °C for 60 s and 95 °C for 15 s. Pomegranate *PgActin* was used as an internal reference gene.

### Data analysis

All data of the anion contents and qRT-PCR were analyzed with one-way ANOVA, and multiple comparisons were evaluated with the Turkey’s test (*p <* 0.05) using the SPSS program (Version 19.0. Chicago, IL, USA) based on the values of three complete randomized blocks. The correlation among variables was analyzed based on the ion content and expressional level of *PgCLCs* and visualized by a ‘corrplot’ package in R [69].

## Supplementary Information

**Additional file 1: Table S1**. A summary of 15 species for phylogenetic analysis and their genomic sources. **Table S2**. List of CLC proteins used for constructing the phylogenetic tree. **Table S3**. Primers used in qRT-PCR analysis for *PgCLCs*. **Table S4**. Dry weights of pomegranate cuttings under NaCl stress. (XLS 67 kb)

**Additional file 2: Fig. S1**. Phylogenetic relationship and conserved motifs of the CLC gene family in land plants. **Fig. S2**. Multiple sequence alignment of all the CLC proteins from 15 species.

## Data Availability

The datasets supporting the conclusions of this article are included within the article (and its additional files).

## Abbreviations

CLC: Chloride channel
CBS: Cystathionine Beta Synthase
Glu: glutamate
pI: Theoretical Isoelectric Point
Mw: Molecular Weight
GRAVY: Grand Average of Hydrophobicity
TMHs: Transmembrane Helices
ML: Maximum Likelihood
WGD: Whole-genome Duplication
OneKP preoject: One Thousand Plant Project
SH-aLRT: Shimodaira-Hasegawa-like aLRT
qRT-PCR: Quantitative real-time polymerase chain reaction
