# Supplementary material for: Genome-wide identification and expression analysis of the CLC gene family in pomegranate (Punica granatum) reveals its roles in salt resistance
Source: BMC Plant Biol. 2020 Dec 11;20:560. doi: 10.1186/s12870-020-02771-z (PMC7733266; doi:10.1186/s12870-020-02771-z)
Supplement: Supplementary file 2 — Additional file 2: Fig. S1. Phylogenetic relationship and conserved motifs of the CLC gene family in land plants. Fig. S2. Multiple sequence alignment of all the CLC proteins from 15 species. [file 12870_2020_2771_MOESM2_ESM.pdf]

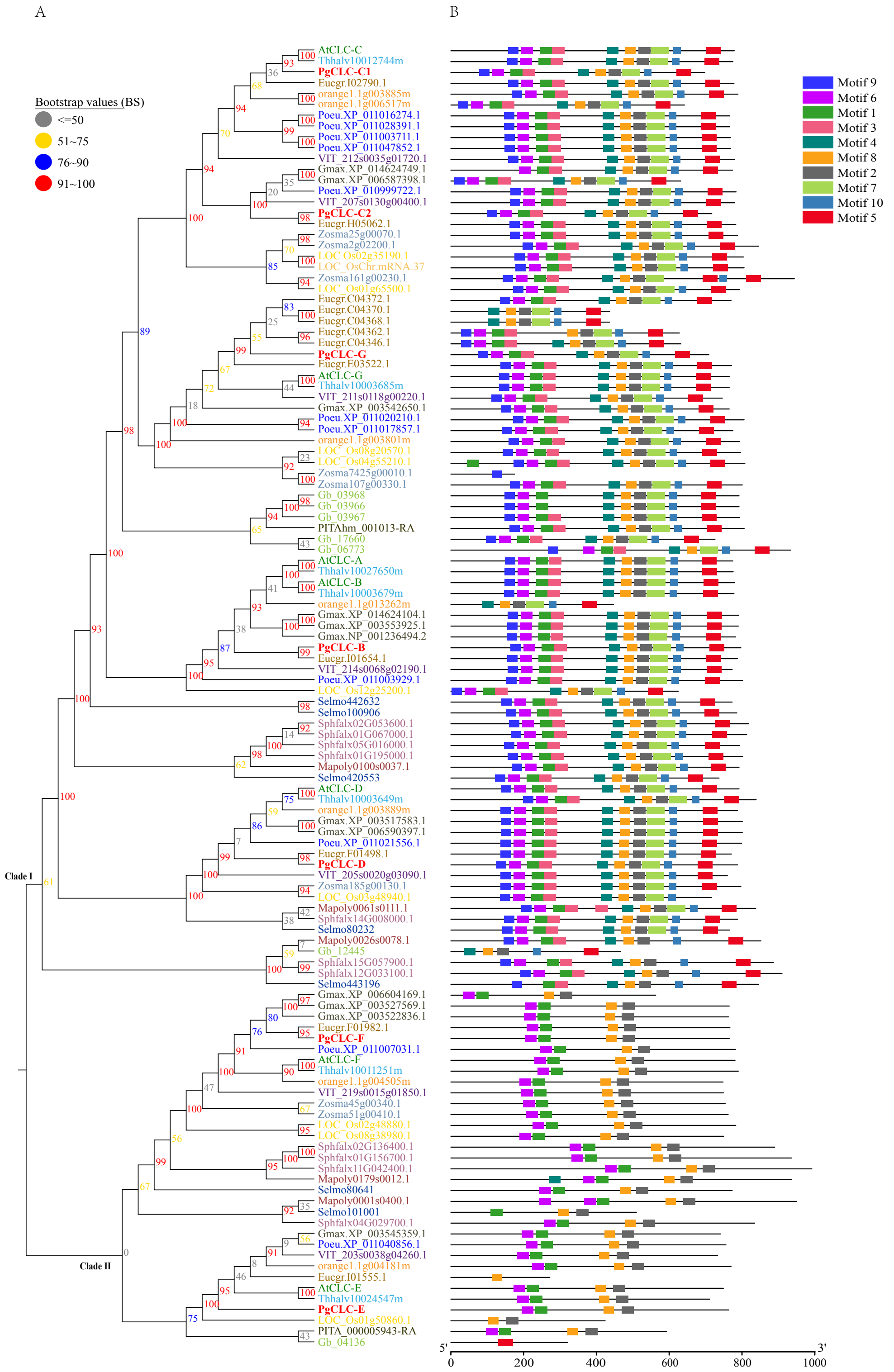

Fig. S1 Phylogenetic relationship and the conserved motifs of the CLC gene family in land plants. **(A)** A phylogenetic tree of the CLC gene family in land plants presented by various branch colors as same as that in Figure 1A. Bootstrap values (BP) are quantified by aLRT statistics with the SH-like procedure. **(B)** Conserved motif distribution of CLC proteins.

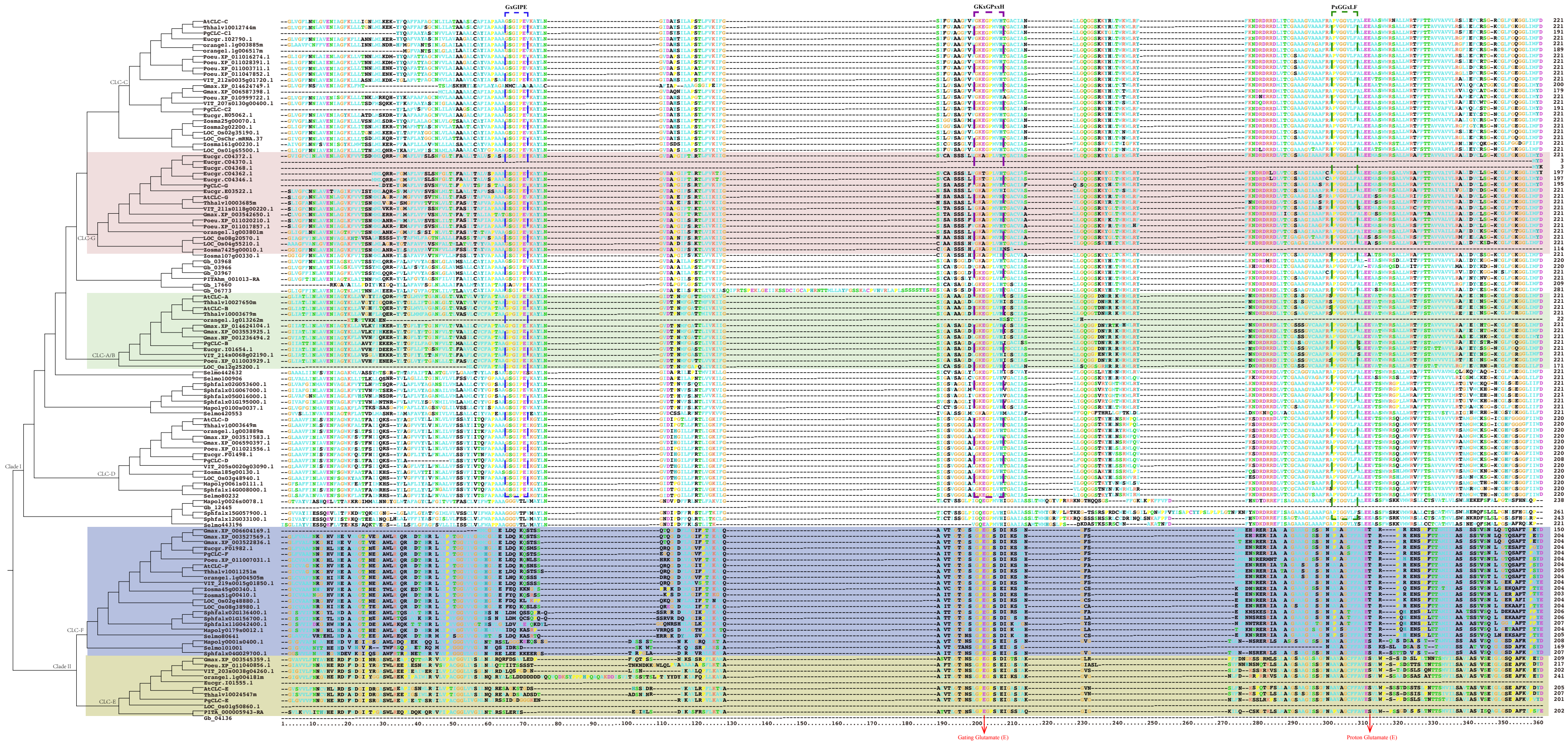

Fig. S2 Multiple sequence alignment of all the CLC proteins from 15 species. The conserved regions: GxGIPE (I), GKxGPxxH (II) and PxxGxLF (III) are circled with dotted boxes in blue, purple and green color, respectively. The conserved gating glutamate (E) and proton glutamate (E) residues are signed in red color at the bottom.
